# Multimodal two-photon imaging using a second harmonic generation-specific dye

**DOI:** 10.1038/ncomms11557

**Published:** 2016-05-09

**Authors:** Mutsuo Nuriya, Shun Fukushima, Atsuya Momotake, Takanori Shinotsuka, Masato Yasui, Tatsuo Arai

**Affiliations:** 1 Department of Pharmacology School of Medicine, Keio University, 35 Shinanomachi, Tokyo 160-8582, Japan; 2 Graduate School of Environment and Information Sciences, Yokohama National University, Yokohama 240-8501, Japan; 3 Graduate School of Pure and Applied Sciences and Tsukuba Research Center for Interdisciplinary Materials Science (TIMS), University of Tsukuba, Tsukuba 305-8577, Japan

## Abstract

Second harmonic generation (SHG) imaging can be used to visualize unique biological phenomena, but currently available dyes limit its application owing to the strong fluorescent signals that they generate together with SHG. Here we report the first non-fluorescent and membrane potential-sensitive SHG-active organic dye Ap3. Ap3 is photostable and generates SH signals at the plasma membrane with virtually no fluorescent signals, in sharp contrast to the previously used fluorescent dye FM4-64. When tested in neurons, Ap3-SHG shows linear membrane potential sensitivity and fast responses to action potentials, and also shows significantly reduced photodamage compared with FM4-64. The SHG-specific nature of Ap3 allows simultaneous and completely independent imaging of SHG signals and fluorescent signals from various reporter molecules, including markers of cellular organelles and intracellular calcium. Therefore, this SHG-specific dye enables true multimodal two-photon imaging in biological samples.

## Abstract (web summary)

Current dyes for second harmonic generation (SHG) imaging strongly fluoresce, limiting their application. Here the authors develop a SHG-specific dye, Ap3, that partitions into cell membranes, displays sensitivity to membrane potential and has virtually no fluorescence emission at SHG imaging wavelengths.

## Graphical abstract

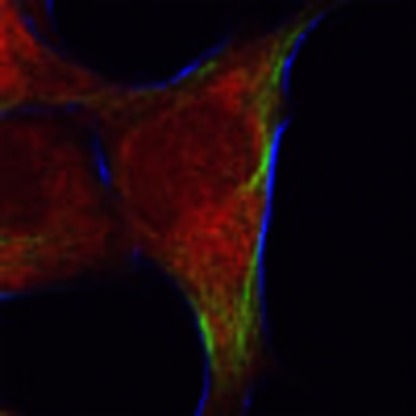

Since the demonstration that two-photon excitation can be achieved by laser-scanning microscopy using ultrashort pulsed lasers^1^, two-photon microscopy has been widely applied to the life sciences^2^,^3^. Two-photon microscopy achieves high-resolution deep-tissue imaging with minimal photobleaching and photodamage outside of the focal spot. However, multi-photon imaging in biological applications generally utilize two-photon excitation of fluorescent molecules, and other multiphoton phenomena are far less frequently employed. A case in point is second harmonic generation (SHG), where two incident photons are converted to a single photon of twice the frequency (half the wavelength) after interacting with materials. In contrast to two-photon excitation, SHG only occurs when the materials lack a centre of symmetry; therefore, SHG imaging can reveal different aspects of molecules (for example, molecular orientation) from those obtained with two-photon excitation microscopy^4^. For example, endogenous proteins, such as collagen and tubulin, form SHG-active structures and are widely utilized for non-label imaging of tissues^5^,^6^,^7^. In addition, SHG-active nanomaterials are being investigated for long-term imaging of these materials *in vivo*^8^. Importantly, SHG imaging can be further extended to functional analyses when SHG-active small exogenous organic dyes are employed. Indeed, dye-based SHG imaging has been shown to be able to specifically report various biological processes, such as membrane potential dynamics^9^,^10^,^11^,^12^ and conformational changes of molecules^13^,^14^,^15^. Despite this unique feature, dye-based SHG imaging has rarely been applied to the life sciences.

A major limitation of dye-based SHG imaging is the lack of dye molecules optimized specifically for SHG imaging that are free of two-photon fluorescence (TPF). Previous studies have utilized pre-existing or newly synthesized molecules^9^,^12^,^16^,^17^,^18^,^19^,^20^,^21^,^22^,^23^,^24^, but most of these emit strong TPF signals. In fact, no dye has been reported to be free of TPF (that is, SHG-specific), which imposes several fundamental limitations. First and foremost, TPF signals originating from SHG chromophores preclude the simultaneous use of other fluorescent probes. Although TPF and SHG are optically separable and can theoretically visualize distinct biological processes simultaneously without any cross-modal interference, such multimodal two-photon imaging cannot be achieved by SHG dyes that emit TPF. Second, unintended photochemical interactions unrelated to SHG can critically compromise the quality of SHG imaging. The disruption of the dye molecules themselves lead to a reduction of SHG signals (that is, photobleaching), and interactions of excited dyes with neighbouring endogenous molecules affect the cellular physiology (that is, photodamage). To avoid these problems and achieve simultaneous multimodal two-photon imaging of SHG and TPF, non-fluorescent SHG-active molecules are needed. Here we report the development and characterization of a novel SHG chromophore Ap3. Ap3 emits no TPF while exhibiting prominent voltage-sensitive SHG signals in cultured cells and brain slices, and shows significantly improved photostability and phototoxicity compared with the previously used SHG dye FM4-64. The SHG-specific nature of Ap3 allows visualization of SHG signals at the plasma membrane without any interference on TPF signals from other fluorescent molecules. Therefore, the SHG-specific Ap3 enables true multimodal two-photon imaging in biological samples.

## Results

### Design, synthesis and characterization of Ap3

**Figure 1:**
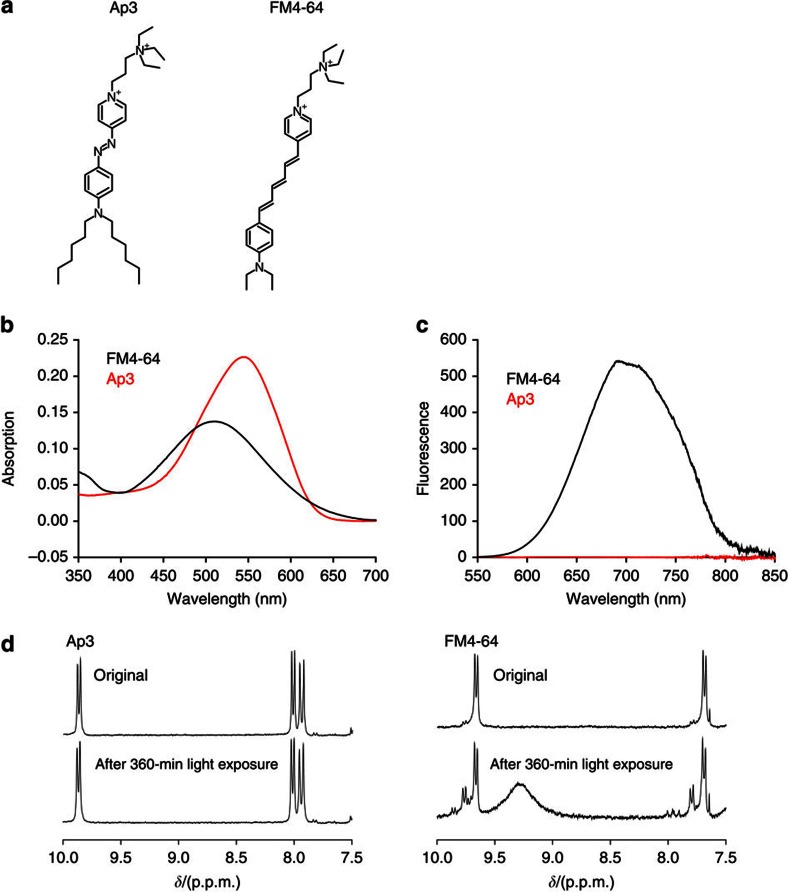
Structure and photochemical properties of Ap3. (**a**) Chemical structures of Ap3 and FM4-64. (**b**) Single-photon absorption spectra of Ap3 (red) and FM4-64 (black) measured at 3.5 μM in 10 mM CHAPS/PBS. (**c**) Fluorescence spectra of Ap3 (red) and FM4-64 (black) measured at 3.5 μM in 10 mM CHAPS/PBS. Excitations were performed at 490 and 500 nm for Ap3 and FM4-64, respectively, to achieve equivalent absorption. (**d**) NMR spectra corresponding to aromatic regions of Ap3 (left) and FM4-64 (right) before and after 360-min light exposure using a 150-W xenon lamp.

In previous studies, hyperpolarizable amphiphilic dyes were successfully used for SHG imaging of plasma membranes^25^. These molecules insert hydrophobic moieties into the membrane, and leave charged hydrophilic parts outside. This helps to orderly align them at the liquid/membrane interface, thus breaking the centre of symmetry and making them ideal SHG materials. Therefore, we focused on molecules that are structurally similar to the widely used SHG dye FM4-64 (refs 18, 26, 27, 28, 29, 30; Fig. 1a), but exhibit non-fluorescent interactions with light. We hypothesized that this goal can be achieved by replacing the chromophore of FM4-64 with an azobenzene group, which undergoes rapid non-radiative deactivation from the electronically excited states via *trans*–*cis* isomerization and is devoid of undesirable photochemical processes^31^,^32^. This may make the molecule non-fluorescent and photostable. Among the dyes synthetized in this scheme, we found that Ap3 is a non-fluorescent, amphiphilic and water-soluble molecule and is therefore a promising candidate SHG-specific dye (Fig. 1a). Ap3 absorbs single photon of ∼540 nm (Fig. 1b and Supplementary Fig. 1) in the presence of a zwitterionic detergent (CHAPS), but it emits virtually no fluorescence, in sharp contrast to FM4-64 (Fig. 1c). We then tested the photostability of Ap3 by exposing it to high-intensity light (150-W xenon lamp) for up to 360 min. This manipulation induced progressive changes in the nuclear magnetic resonance (NMR) spectra of FM4-64, suggesting the susceptibility of FM4-64 to photochemical reactions and therefore photo-instability (Fig. 1d and Supplementary Fig. 2). In contrast, no change was observed in the NMR spectra or high-performance liquid chromatography patterns of Ap3 under the same conditions, showing the highly photostable nature of Ap3 (Fig. 1d and Supplementary Figs 2 and 3).

### SHG-specificity of Ap3

**Figure 2:**
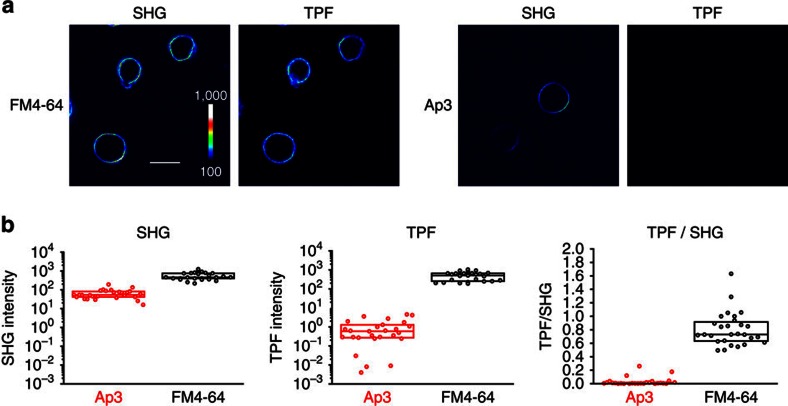
SHG-specificity of Ap3. (**a**) Representative SHG and TPF signals obtained from cultured CHO cells loaded with 20 μM FM4-64 (left) and Ap3 (right). The signal intensity is shown in pseudocolour. Scale bar, 20 μm. (**b**) Quantitative analysis of SHG and TPF signals. Signals of SHG (left) and TPF (middle) from the plasma membranes of CHO cells stained with Ap3 (red) and FM4-64 (black) were quantified and summarized. Individual points indicate data from each cell (*n*=27 cells for Ap3 and 28 cells for FM4-64) and boxes indicates the 25th, 50th and 75th percentile values.

The potential application of Ap3 in SHG imaging was tested using Chinese hamster ovary (CHO) cells, with FM4-64 dye as a reference, using a multi-photon microscopy system (Supplementary Fig. 4). These dyes were added to extracellular solution at a final concentration of 20 μM, and two-photon imaging was performed using a femtosecond laser tuned to 950 nm, which effectively excites various fluorescent molecules^33^,^34^. When illuminated, FM4-64 generates strong SHG and TPF signals (Fig. 2a). In contrast, while Ap3 is readily adsorbed onto the plasma membrane and generates strong SHG signals (Supplementary Fig. 5), no TPF signals were observed (Fig. 2a), consistent with the single-photon data (Fig. 1c). Quantitative analysis revealed that the TPF signal relative to the SHG signal (TPF/SHG) was practically zero for Ap3 and was significantly lower than that of FM4-64 (Fig. 2b, *P*<5 × 10^−9^, Mann–Whitney test). SHG signals remain stable for tens of minutes under this experimental condition, and then exhibit a gradual decrease over time, presumably due to the flip–flop movement of Ap3 from the outer to inner leaflet of the lipid bilayer, resulting in disruption of the non-centrosymmetric distribution of Ap3 molecules at the plasma membrane (Supplementary Fig. 5) (ref. 35). Thus, Ap3 serves as an SHG-active dye that is devoid of TPF in cultured cells, in sharp contrast to FM4-64.

### Characterizations of Ap3-SHG in neurons

**Figure 3:**
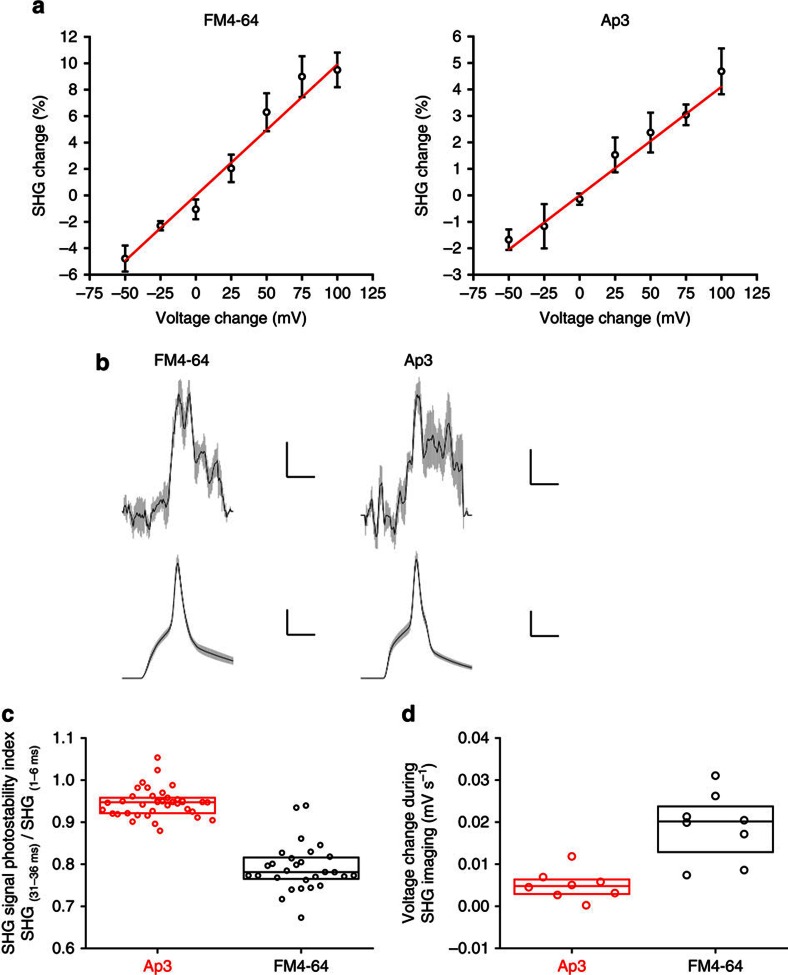
Membrane potential sensitivity, photostability and phototoxicity of Ap3-SHG assessed in cortical neurons. (**a**) Membrane potential sensitivity of SHG signals from FM4-64 and Ap3 assessed by voltage clamp combined with point scan in cortical neurons. Shown are SHG signal changes at each voltage step from a holding potential of −65 mV as means±s.e.m. (black, *n*=28 points from 4 cells for FM4-64 and 38 points from 8 cells for Ap3), together with linear fit curves (red). (**b**) SHG signal changes on action potential in neurons. SHG signals were monitored by a point-scan protocol at the soma of patch-clamped neurons in brain slices (scale bars, 2% and 10 ms for FM4-64 and 1% and 10 ms for Ap3) with simultaneous electrophysiological recording (scale bars, 20 mV and 10 ms). Data are shown as means (black)±s.e. m. (light grey) (*n*=5 cells for FM4-64 and 6 cells for Ap3). (**c**) Photostability of Ap3-SHG signals in neurons. The SHG signal photostability index was calculated by comparing the mean SHG signal intensities at the initial 5 ms and those 30 ms later during 40-ms-long continuous laser illumination. Individual data points (circles) are shown with boxes corresponding to the 25th, 50th and 75th percentile (*n*=38 cells for Ap3 an 28 cells for FM4-64). (**d**) Reduced phototoxicity of Ap3 SHG imaging in neurons. Neurons in brain slices were loaded with Ap3 (500 μM) or FM4-64 (200 μM) and frame-scan imagings was performed with a long pixel dwell time (100 μs per pixel) to induce photodamage while monitoring membrane potential changes. Individual data points (circles) are shown with boxes corresponding to the 25th, 50th and 75th percentile (*n*=8 for both Ap3 and FM4-64).

Previous studies have reported that SHG signals from various molecules are linearly sensitive to membrane potential changes^11^,^12^,^16^,^18^,^22^,^26^,^36^. As SHG is membrane selective, all SHG signals collected from the focal point are sensitive to membrane potential changes and are free of background membrane potential-insensitive signals as is the case for all fluorescence-based probes. This allows the measurement of membrane potential dynamics in cellular structures, such as dendritic spines and axons, that are otherwise difficult to access^26^,^28^,^29^,^30^. Therefore, we tested whether Ap3-derived SHG signals are sensitive to changes in membrane potential using a point-scan protocol, where the laser is fixed to a single point of interest and the SHG signal is collected continuously^26^,^28^. First, membrane potential sensitivity was examined for FM4-64 and Ap3 using a voltage-clamp protocol. Both showed linear responses to a wide range of voltage changes with a sensitivity of 9.9±0.6% per 100 mV for FM4-64 and 4.1±0.2% per 100 mV for Ap3 (Fig. 3a, mean±s.e.m., *R*=0.99 for both FM4-64 and Ap3). Next, SHG signals were measured while inducing action potentials in neurons under a current-clamp protocol. Ap3-SHG signals changed quickly on membrane potential changes and showed minimal delays (∼0.3 ms) when the peak time was compared, similar to FM4-64 (Fig. 3b). During point-scan imaging, time-dependent reductions in SHG signals were observed (Supplementary Fig. 6), presumably due to photobleaching of the dye molecules. To compare the stability of SHG signals, the degrees of SHG signal decay during point scans were compared for Ap3 and FM4-64. Consistent with the photostability of Ap3 assessed in solution (Fig. 1d and Supplementary Figs 2 and 3), the SHG signal photostability was significantly higher for Ap3 compared with FM4-64 (Fig. 3c, *P*<1 × 10^−10^, Mann–Whitney test). These results further raise the possibility that the photostable nature of Ap3 may circumvent the unintended photochemical reactions that then become toxic to neurons. To test this possibility, neurons were imaged in frame-scan mode with a long pixel dwell time (100 μs per pixel, instead of 2 μs per pixel used in regular frame scan imagings) to intentionally induce photodamage. Indeed, this imaging protocol induced time-dependent membrane potential depolarization in neurons loaded with FM4-64, as reported previously^30^. However, in the condition that result in indistinguishable SHG signal intensities, the photodamage was significantly reduced in Ap3-SHG imaging (Fig. 3d and Supplementary Fig. 7, *P*<2 × 10^−3^, Mann–Whitney test). Taken together, these results demonstrate that Ap3 is a photostable and less phototoxic membrane potential-sensitive SHG-specific dye that can be applied to cultured cells as well as neurons in brain slices.

### Multimodal two-photon imaging of cellular structures

**Figure 4:**
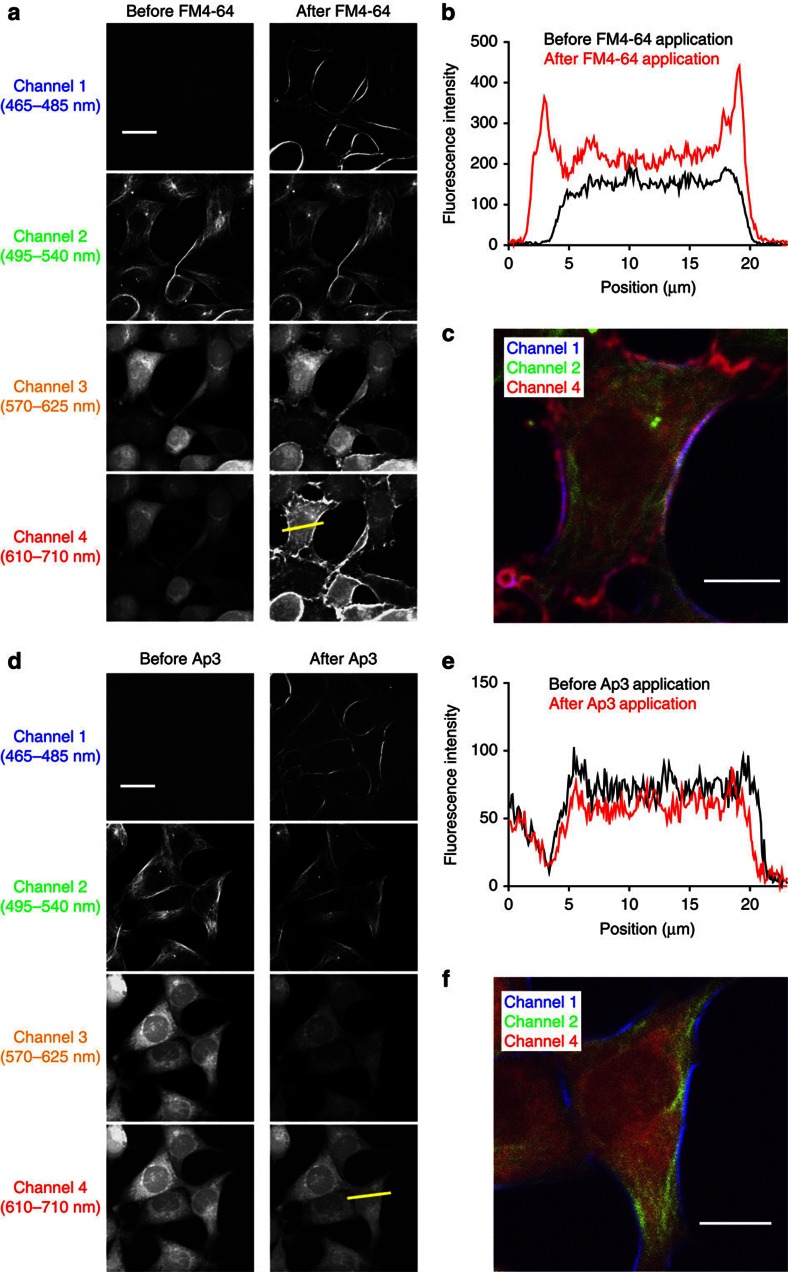
Multimodal two-photon imaging of cellular structures in CHO cells. Cultured CHO cells were loaded with Tubulin Tracker Green and Calcein Red-Orange, and *Z*-stack images were taken before and after the application of FM4-64 or Ap3. (**a**,**d**) SHG and TPF signals before and after the application of SHG dyes. SHG signals (channel 1) as well as TPF signals in three different spectral ranges (channels 2–4) are shown separately. Scale bar, 20 μm. (**b**,**e**) Intensity profiles of TPF signals in channel 4 before (black) and after (red) the application of SHG dyes, at the yellow lines indicated in **a** and **d**. (**c**,**f**) Merged images of channels 1, 2 and 4 after the application of SHG dyes. Scale bar, 10 μm.

Having confirmed the SHG-specific nature of Ap3, we explored the possibility of multimodal two-photon imaging by combining it with other fluorescent molecules. First, considering the wide use of FM4-64 as a plasma membrane marker, simultaneous imaging of SHG with other fluorescent structural markers was examined. CHO cells were loaded with various types of fluorescent cell marker molecules and visualized by two-photon microscopy before and after the application of SHG dyes (Fig. 4 and Supplementary Figs 8 and 9). The addition of FM4-64 to the imaging solution not only generated SHG signals in the blue wavelength range (channel 1) but also generated strong fluorescence signals spanning a wide range of wavelengths in the yellow–red region (channels 3 and 4, Fig. 4a and Supplementary Figs 8a and 9a). This crossover into the yellow–red channels significantly interfered with the accurate detection of the red dye, the cytoplasmic marker Calcein Red-Orange (Fig. 4b), and generated erroneously overlapping signals of SHG and TPF in the merged image (Fig. 4c). In sharp contrast, the application of Ap3 did not alter the spatial patterns of pre-existing fluorescence signals, while generating SHG signals at the plasma membrane (Fig. 4d,e and Supplementary Figs 8b and 9b). This allowed completely isolated, simultaneous imaging of signals from multiple modalities (Fig. 4f). Note that general reductions in the fluorescence signals were observed invariably following the addition of chromophores (Supplementary Fig. 10).

### Functional multimodal two-photon imaging in neurons

**Figure 5:**
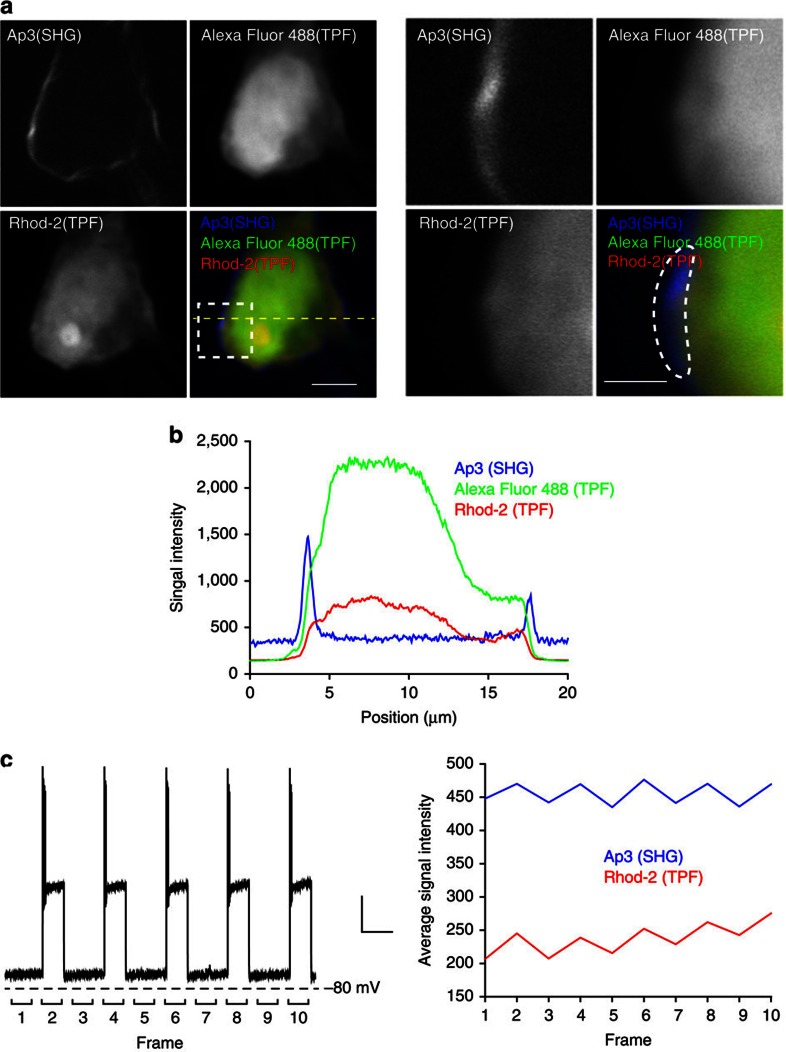
Multimodal two-photon imaging of membrane potential and intracellular calcium dynamics in cortical neurons. (**a**) Representative images of Ap3, Alexa Fluor 488 and Rhod-2 at the soma of a patch-clamped neuron. SHG signals from Ap3 were simultaneously obtained with two-photon fluorescence signals of freely diffusible Alexa Fluor 488 dye, as well as the calcium indicator Rhod-2. Membrane proximal region in dotted square (left) is enlarged in (right). Scale bars, 5 μm (left) and 2 μm (right). (**b**) Intensity profiles of Ap3-SHG (blue), Alexa Fluor 488-TPF (green) and Rhod-2-TPF (red) at the line indicated by the yellow dotted line in **a**, left. (**c**) Simultaneous imaging of membrane potential and calcium concentration at the membrane proximity region indicated in the white dotted region in **a**, right. Frame scan was performed at 0.2 Hz with current injection in even-numbered frames that evoke depolarization (left). Scale bars, 20 mV and 5 s. Quantification of simultaneously obtained Ap3-SHG (blue) and Rhod-2-TPF (red) signals show membrane potential and intracellular calcium concentration changes near the membrane (right).

Taking advantage of the multimodal two-photon imaging ability of Ap3, we then attempted to simultaneously monitor changes in membrane potential and calcium concentration in the cytoplasm. To this end, neurons in acute brain slices were patch-clamped and loaded with the fluorescent calcium indicator Rhod-2 together with Ap3, as well as Alexa Fluor 488, which was used as a morphology marker. Simultaneous imaging reveal that two-photon fluorescent signals in green (Alexa Fluor 488) and red (Rhod-2) channels are strictly restricted to inside the cell and do not contain those from the plasma membrane, while SHG signals in the blue channel arise only from the plasma membrane (Fig. 5a,b). Importantly, fluorescence patterns of Alexa Fluor 488 and Rhod-2 in Ap3-loaded neurons were indistinguishable from those without Ap3, while the inclusion of FM4-64 in the pipette resulted in strong fluorescence from FM4-64 and significantly altered fluorescence signal patterns in the red channel (Supplementary Fig. 11). These results confirmed the capacity for completely independent multimodal two-photon imaging of Ap3 in tissue samples. Then, multimodal two-photon imaging was performed in the frame-scan mode while alternatively applying a current injection that depolarizes the neuron with concomitant action potentials in every other frame (Fig. 5c). When the membrane and juxtamembrane region was selected for analysis (dotted region in Fig. 5a right; average width 0.94 μm), the change in calcium concentration at this region was successfully revealed by Rhod-2 TPF together with the membrane potential change by Ap3-SHG (Fig. 5c). Therefore, multimodal two-photon imaging can monitor membrane potential changes together with biochemical changes of the juxtamembrane region simultaneously without any cross-modal signal interference.

## Discussion

In this study, we successfully developed an SHG-specific dye, Ap3, and applied it to multimodal two-photon imaging in cultured cells as well as neurons in acute brain slices. Ap3 is distinct from other organic dyes currently available for cellular imaging, including FM4-64, owing to its SHG specificity and serves as a prototype for further SHG dye development.

The repertoire of fluorescent reporters of various biological phenomena is continually expanding both for small synthetic dyes and proteins^37^,^38^. In contrast, the development of organic dyes specifically designed for SHG has not been actively pursued. Using a limited number of SHG-active fluorescent molecules, however, dye-based SHG imaging has shown great promise in the visualization of various biological phenomena, including membrane potential, membrane transport, lipid domain and other processes occurring at the membrane^39^,^40^,^41^,^42^. Furthermore, labelling with SHG-active dyes has been utilized to measure conformational changes of target molecules, such as proteins and DNA^13^,^14^,^15^,^43^, indicating further opportunities for the application of dye-based SHG imaging. The photostable and SHG-specific nature of Ap3 should facilitate its applications to all of the aforementioned uses. In addition, the SHG-specificity of Ap3 allows completely independent imaging of SHG and fluorescent signals at the same time. Therefore, when combined with fluorescent probes, this new technology will enable the monitoring of multiple different events simultaneously from the same focal spot.

In this study, we utilized Ap3 as a membrane marker that allows multimodal two-photon imaging with other fluorescent markers and applied it to cultured CHO cells and neurons in acute brain slices. Previous studies have shown that the juxtamembrane region has specific biochemical reactions including calcium microdomains^44^,^45^. Using Ap3, clear identification of plasma membrane structures and measurement of voltage changes can be achieved together with simultaneous monitoring of biochemical reactions using fluorescent indicators from single-laser illumination, without the risk of cross-modal signal interactions. In sharp contrast, previously utilized SHG dyes, such as FM4-64, emit strong fluorescent signals; therefore, analyses of other markers become inaccurate. Indeed, although qualitative analyses of voltage and calcium fluctuations have been successfully performed using fluorescent reporters^46^, spectral overlaps between dyes necessitate sub-optimal signal acquisitions, as well as post-processing steps to effectively separate signals. In addition, our results demonstrate that Ap3 is more photostable and less phototoxic in cellular imagings. Therefore, Ap3-SHG imaging is expected to be a simple and powerful tool to characterize the biophysical and biochemical properties of cellular microdomains at and near the plasma membrane.

To improve organic dye-based SHG imaging, it is important to consider some theoretical aspects. As Ap3 is expected to undergo rapid non-radiative deactivation from the excited state to the ground state^31^,^47^, we cannot assess the two-photon excitation of Ap3 that may take place in addition to SHG. As such, it remains possible that Ap3 in the excited state contributes to the generation of SH signals, in addition to that in the ground state. Similarly, the sensitivity of SHG signals to voltage is likely to depend on the wavelength of the laser^12^,^48^,^49^. Although it is beyond the scope of this study, a detailed characterization of nonlinear responses of Ap3 to a wide spectrum range of ultrashort pulse lasers is expected to provide additional clues that are important for the future development of SHG-specific dyes and optimization of SHG imaging conditions.

## Methods

### Materials

All reagents and solvents used for the synthesis and spectroscopic measurements were of the highest commercial quality and were used without purification. Tetrahydrofuran (THF), dimethyl sulfoxide, methanol, distilled water, acetic acid, 4-aminopyridine, tetrafluoroboric acid, sodium nitrite, *N,N*-dihexylaniline and CHAPS (3-[(3-cholamidopropyl)dimethylammonio]propanesulfonate) were purchased from Wako Pure Chemical Industries. Ethyl acetate was purchased from Kanto Chemical. FM4-64 was obtained from Biotium (SynaptoRed C2). All other reagents were purchased from Tokyo Chemical Industry or Sigma Aldrich, unless otherwise noted.

### Chemical synthesis and characterization

NMR spectra were recorded on a JNM-EX270 (^1^H NMR at 270 MHz; ^13^C at 67.8 MHz) or AV-400FT NMR (^1^H NMR at 400 MHz; ^13^C at 100 MHz). *δ* values are given in parts per million relative to the peak for tetramethylsilane. Silica gel column chromatography was performed using silica gel 60N (Kanto Chemical) or Wakogel 50NH_2_ (Wako Pure Chemical Industries). High-resolution mass spectra (HRMS) were measured on a JEOL AccuTOF CS. Absorption spectra were measured on a Shimadzu UV-1600. All absorption measurements were carried out at room temperature under air. See Supplementary Fig. 12 for the schematic illustration of the chemical synthesis of Ap3.

*Compound 1*. a solution of 4-aminopyridine (198 mg, 2.0 mmol) and 42% tetrafluoroboric acid (5 g) was cooled to 0 °C in an ice bath and stirred to give a milky solution. Sodium nitrite (138 mg, 2.0 mmol) dissolved in 5 ml of water was added slowly to the mixture under efficient stirring with at a constant temperature of 0 °C. *N,N*-dihexylaniline (1.3 g, 5.0 mmol) in 3 ml of acetic acid was slowly added to the mixture under efficient stirring. Then, 5 ml of THF was added to the mixture for homogeneous mixing and was stirred for 3 h while slowly raising the temperature to room temperature. After the reaction was completed, 50 ml of 2 M sodium hydroxide was added to the reaction mixture and was extracted with ethyl acetate. The organic layer was dried over Na_2_SO_4_, filtered and evaporated. The residue was purified with column chromatography (SiO_2_, eluent: dichloromethane:methanol=15/1, v/v), which provided a red oil as the product (220 mg, 0.6 mmol) with 30% yield.

^1^H NMR(CDCl_3_, 270 MHz) *δ* 0.91 (t, *J*=6.7 Hz, 6H). 1.28–1.43 (m, 12H), 1.55–1.71 (m, 4H), 3.37 (t, *J*=7.7 Hz, 4H), 6.68 (dd, *J*_1_=1.9 Hz, *J*_2_=6.3 Hz, 2H), 7.62 (dd, *J*_1_=1.6 Hz, *J*_2_=4.6 Hz, 2H), 7.87 (dd, *J*_1_=1.9 Hz, *J*_2_=6.3 Hz, 2H), 8.70 (dd, *J*_1_=1.6 Hz, *J*_2_=4.6 Hz, 2H); ^13^C NMR(CDCl_3_, 67.5 MHz) *δ* 14.0, 22.6, 26.7, 27.3, 31.6, 51.3, 111.1, 116.0, 126.2, 143.0, 150.0, 151.6, 158.1; HRMS (ESI) *m* *z*^−1^ calculated for C_23_H_34_N_4_ [M+H]^+^: 367.2856, found 367.2856.

*Ap3*. A mixture of compound 1 (37 mg, 0.1 mmol) and (3-bromopropyl)triethylammonium bromide (30 mg, 0.1 mmol) in acetonitrile was stirred at 80 °C under nitrogen atmosphere. The solution turned dark purple during the reaction. After the reaction proceeded for 24 h, the solvent was removed on a rotary evaporator. The dark purple residual oil was washed with hexane and ether. Purification by column chromatography (SiO_2_-amino), followed by elution with a gradient solvent system of dichloromethane:methanol (from 100/1 to 15/1, v/v) was performed three times, yielding 1.0 mg (2%) of Ap3 as a deep purple oil. The resulting Ap3 dye was dissolved in the internal solution containing 10 mM NaCl, 10 mM KCl, 135 mM KMeSO_4_, 2.5 mM MgATP, 0.3 mM NaGTP and 10 mM HEPES (pH 7.3) at a concentration of 10 mM.

^1^H NMR(CDCl_3_, 400 MHz) *δ* 0.92 (t, *J*=6.7 Hz, 6H). 1.25–1.42 (m, 12H), 1.50 (t, *J*=7.0 Hz, 9H), 1.60–1.77 (m, 4H), 2.83–2.98 (m, 2H), 3.43-3.50 (m, 10H), 3.83 (t, *J*=8.0 Hz, 2H), 5.18 (t, *J*=7.8 Hz, 2H), 6.74 (d, *J*=9.4 Hz, 2H), 7.94 (d, *J*=9.4 Hz, 2H), 8.01 (d, *J*=6.8 Hz, 2H), 9.88 (d, *J*=6.8 Hz, 2H); ^13^C NMR(CDCl_3_, 100 MHz) 8.4, 14.0, 22.6, 25.9, 26.7, 27.6, 31.5, 51.8, 54.2, 54.7, 55.7, 112.3, 118.9, 144.7, 146.2, 154.5, 162.4; HRMS (ESI) *m* *z*^−*1*^ calculated forC_32_H_55_N_5_ [M]^2+^: 254.7223, found 254.7216.

### Ultraviolet–vis spectroscopy

The absorption spectra of Ap3 and FM4-64 in phosphate-buffered saline (PBS, pH 7.4) containing 10 mM CHAPS, THF, dimethyl sulfoxide, methanol and distilled water were measured at a 3.5-μM concentration using a Shimadzu UV-1600 ultraviolet–visible spectrophotometer. All absorption measurements were carried out in a 1-cm path length quartz cuvette at room temperature under ambient atmosphere.

### Fluorescence spectroscopy

Fluorescence spectra of Ap3 and FM4-64 in PBS (pH 7.4) containing 10 mM CHAPS were measured using a Hitachi F-7000 fluorescence spectrophotometer at a 3.5-μM concentration with excitation and emission slit widths both set to 10 nm. The excitation wavelengths for Ap3 and FM4-64 were 490 and 500 nm, respectively. The absorbance values at the excitation wavelengths were the same for Ap3 and FM4-64 under this condition. All fluorescence measurements were carried out in a 1-cm path length quartz cuvette at room temperature under ambient atmosphere.

### Photostability analysis in solution

The NMR solutions of Ap3 or FM4-64 in CDCl_3_ in a Pylex NMR tube were irradiated at 25 °C for 0, 10, 60, 120 and 360 min using a 150-W xenon lamp (Ushio UXL-159) with a monochromator. The irradiation wavelengths were set to 570 and 500 nm for Ap3 and FM4-64, respectively, which are the absorption maxima of these dyes in chloroform.

In addition to NMR, Ap3 was also analysed by high-performance liquid chromatography using methyl orange as an internal standard. Ap3 solution was irradiated at 600 nm, which is absorbed by Ap3 but not by methyl orange, and detected at 500 nm light, which is absorbed both by Ap3 and methyl orange. The eluent was acetonitrile:water (60:40) containing 0.2% (v/v) trifluoroacetic acid.

### Cell culture

CHO cells (a kind gift from Dr Yu at Keio University, originally obtained from American type culture collection and not tested for mycoplasma infection) were maintained in culture media (10% fetal bovine serum in Dulbecco's modified Eagle's medium supplemented with penicillin and streptomycin) in a humidified 5% CO_2_ incubator at 37 °C until use. For imaging, CHO cells were plated on poly-L-lysine (100 μg ml^−1^ in 0.1 M borate buffer, pH 8.5)-coated coverslips (Fisher Scientific, ϕ12 mm, ∼0.15 mm thickness) in 35-mm culture dishes (Falcon) or glass-bottom dishes (Iwaki, ϕ12 mm, ∼0.15 mm thickness) coated in the same manner at a density of 5 × 10^4^ cells per 35-mm dish.

### Dye loading of CHO cells

Stock solutions of Ap3 and FM4-64 were diluted in the imaging buffer (125 mM NaCl, 5 mM KCl, 10 mM dextrose, 10 mM HEPES, 1 mM MgCl_2_ and 2 mM CaCl_2_, pH 7.3) and applied to the cells at a final concentration of 10–20 μM. Both dyes were readily adsorbed to the membrane and reached equilibrium within minutes, without any obvious cellular toxicity during the imaging period (Supplementary Fig. 5). For multimodal imaging experiments, pHrodo Green Dextran (Life Technologies, 20 μg ml^−1^), Calcein Red-Orange (Life Technologies, 0.5 μM), Tubulin Tracker Green (Life Technologies, 0.5 μM), ER-Tracker Red (Life Technologies, 5 μM) and Calcein (Life Technologies, 0.5 μM) were applied to cells in the imaging buffer at the indicated concentration and incubated at 37 °C for 30 min–1 h before imaging. For multimodal imaging with EGFP and DsRed, a total of 1.5–2.0 μg of pEGFP-N1 and pDsRed-N1 empty vectors was transfected into CHO cells using 3.0–4.0 μl of Lipofectamine 2000 (Life Technologies) following the manufacturer's protocol, and imaging was performed 24–60 h after transfection.

### Two-photon imaging of CHO cells

The culture medium was replaced by imaging buffer before imaging, which was performed using FV10 software (Olympus) on an FV1000MPE multiphoton microscopy system (Olympus) equipped with MaitaiHP Ti:sapphire mode-locked femtosecond laser working at repetition rates of 80 MHz (Newport) and a LUMPlanFL60 × WIR2 objective lens (numerical aperture, 0.9, working distance, 2 mm; Olympus; Supplementary Fig. 4). SHG signals generated from 950-nm laser illumination were collected using an external photomultiplier tube (PMT) located at the bottom of the sample (Olympus) after a 465–485 nm band pass filter (channel 1). The typical laser power used for SHG dye imaging (final concentration, 10–20 μM) was 8–12 mW under an objective lens. For simultaneous two-photon fluorescence imaging, fluorescent signals were simultaneously collected using external PMTs after band pass filters 495–540 (channel 2), 570–625 (channel 3) and 610–710 nm (channel 4). For a quantitative comparison of SHG and TPF signals from Ap3 and FM4-64 (Fig. 2), the same concentrations of dyes were applied to cells and images were taken in channel 1 (SHG signal) and channel 4 (TPF signal) in the exact same manner, including laser power and acquisition parameters. Similarly, in experiments examining the effect of SHG dyes on fluorescence signals from other molecules (Supplementary Fig. 10), images were taken with all parameters fixed throughout the experiments. In these experiments, concentrated dye solutions (FM4-64, Ap3, Alexa Fluor 594 Hydrazide, DiI, FM1-43 and compound 1 in our previous report^23^, all 200 μM in imaging buffer) were directly applied to the imaging buffer covering the sample coverslips and mixed well to achieve final concentrations indicated. All recordings were performed at room temperature. Data were collected in a randomized order to avoid any systematic changes among groups.

### Neuronal loading and patch-clamp recording

All animal experiments were performed according to the Guidelines for the Care and Use of Laboratory Animals of Keio University. The 300-μm-thick cortical slices were prepared from 2- to 3-week-old C57/BL 6J mice of either sex. Neurons were visualized and patch clamped under IR-DIC using a micromanipulator (EMM-3SV, Narishige) in a recording chamber that was perfused with artificial cerebrospinal fluid containing 126 mM NaCl, 3 mM KCl, 1.14 mM NaH_2_PO_4_, 26 mM NaHCO_3_, 3 mM CaCl_2_, 1 mM MgCl_2_ and 10 mM dextrose (pH 7.4), bubbled with 95% O_2_/5% CO_2_. In the voltage-clamp experiments, 1 mM NiCl_2_ and 1 μM tetrodotoxin were included in the artificial cerebrospinal fluid to block voltage-gated calcium channels and sodium channels, respectively. Glass pipettes (5–10 MΩ; Warner Instruments) were filled with 100–400 μM Ap3 or 100–200 μM FM4-64 together with 100 μM Alexa Fluor 488 hydrazide (Life Technologies) in the internal solution containing 10 mM NaCl, 10 mM KCl, 135 mM KMeSO_4_, 2.5 mM MgATP, 0.3 mM NaGTP and 10 mM HEPES (pH 7.3), and neurons were loaded under the whole-cell patch-clamp configuration using MultiClamp 700B (Molecular Devices). In the multimodal two-photon imaging with a calcium indicator, Rhod-2 tripotassium salt (Life Technologies, 100 μM) was also included in the internal solution. All recordings were performed at room temperature.

### Two-photon imaging of neurons

Neurons loaded with Ap3 or FM4-64 were illuminated using a 950-nm laser, and TPF and SHG signals were simultaneously collected. In the frame-scan mode, the membrane potential of neurons was manipulated frame by frame in a sequential manner under current clamp (Fig. 5c) using custom-made software (LabVIEW, National Instruments) at 0.2 Hz. In the point-scan recordings, 40-ms-long laser pulses were applied to the target position at 2 Hz up to 100 times using FV10ASW multipoint-scan module (Olympus), and voltage or current pulses were delivered to neurons in every other laser pulse with a 10-ms delay after the start of laser illumination. SHG signals from the PMT were continuously recorded by a data acquisition board (National Instruments) together with electrophysiological signals at 3 kHz using custom software (LabVIEW, National Instruments) after 1 kHz low pass filter using an isolation amplifier (NF Corporation). Data were collected in a randomized order to avoid any systematic changes among groups.

### Phototoxicity assay in neurons

Phototoxicity assays of Ap3 and FM4-64 in neurons were performed in accordance with a previous report^30^. To induce photodamage to neurons, the neurons were loaded with Ap3 (500 μM) or FM4-64 (200 μM) and imaged in frame-scan mode with a 50-times-longer pixel dwell time (100 μs per pixel) than used for normal scan (2 μs per pixel). Frame scanning was performed at the somatic area continuously for up to 30 frames while the membrane potential was monitored in the current-clamp configuration, and the slopes of depolarization obtained from linear regressions were used as an index of photodamage. Laser intensities were adjusted to obtain comparable SHG signals at the soma under the same image acquisition settings.

### Data analysis

Quantification of the TPF and SHG signal intensities from CHO cells was performed using ImageJ software (NIH) by calculating mean signal intensities at the plasma membrane from each cell. Sensitivity of the SHG signal to the membrane potential of neurons was analysed using FV10 software and ImageJ; the average SHG signals were obtained from the pixels inside the regions of interest that were manually selected to cover the plasma of the soma (as visualized under SHG imaging). SHG and TPF intensity profiles before and after the application of SHG chromophores were generated using the ImageJ ‘plot profile' function. In all figures describing differences between groups (that is, before and after SHG dye application and membrane potential differences), images taken under two different conditions are displayed on the same linear scale for comparison. In the point-scan experiments, average data from control and stimulated groups were normalized using the initial 10-ms period, when no stimulation was delivered to compensate for any systematic changes, and changes in current, voltage and SHG signals were calculated using custom software (MATLAB). To calculate voltage sensitivity, average SHG signal changes at 5–15 ms after the onset of voltage pulses were used. SHG signal intensity data under control conditions in point-scan mode were used to calculate the extent of photobleaching. Statistical analyses were performed using Origin Pro (OriginLab) using non-parametric Mann–Whitney tests because data points were not normally distributed when assessed by the Shapiro–Wilk test.

## Additional information

**How to cite this article**: Nuriya, M. *et al*. Multimodal two-photon imaging using a second harmonic generation-specific dye. *Nat. Commun.* 7:11557 doi: 10.1038/ncomms11557 (2016).

## Supplementary Material

Supplementary Information Supplementary Figures 1-12

Peer review file
