# Supplementary material for: Multimodal two-photon imaging using a second harmonic generation-specific dye
Source: Nat Commun. 2016 May 9;7:11557. doi: 10.1038/ncomms11557 (PMC4865818; doi:10.1038/ncomms11557)
Supplement: Supplementary Information — Supplementary Figures 1-12 [file ncomms11557-s1.pdf]

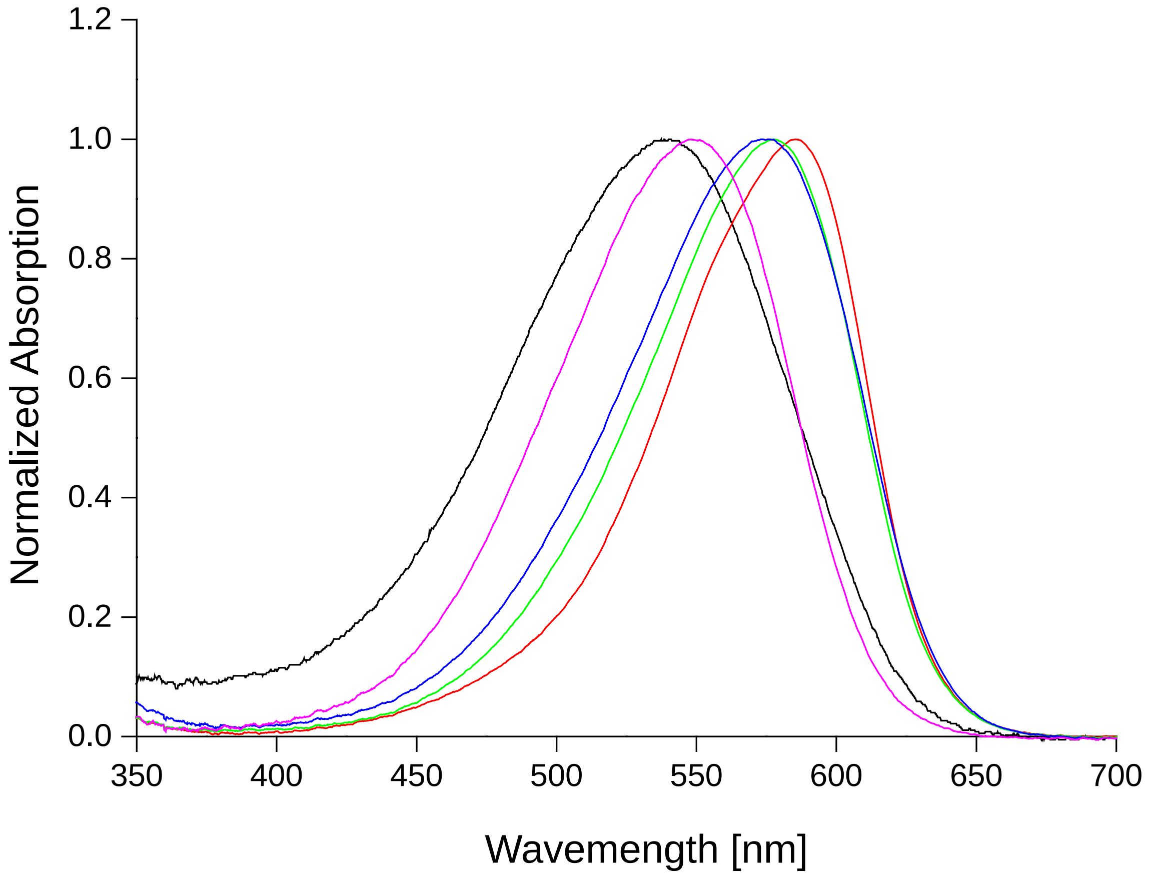

Supplementary Figure 1: Absorption spectra of Ap3 in various solvents.

Absorption spectra of Ap3 in the presence of 10 mM CHAPS in PBS at pH 7.4 (black); THF (magenta); DMSO (blue); methanol (green); distilled water (red).

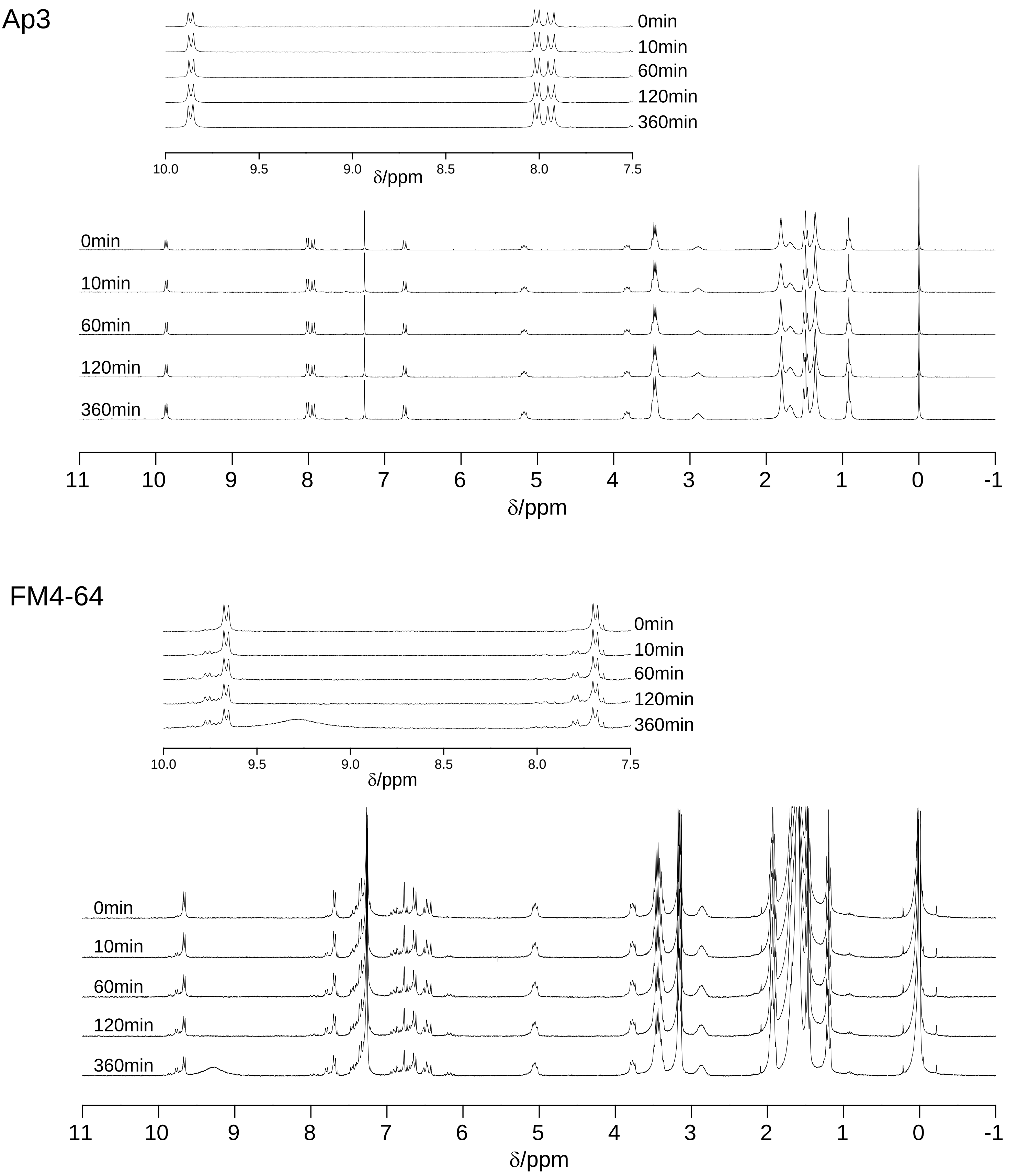

Supplementary Figure 2: Photostability of Ap3 and FM4-64 measured by NMR.

The NMR solutions of Ap3 or FM4-64 in $CDCl_3$ in a Pylex NMR tube were irradiated at 298 K for 0, 10, 60, 120, and 360 minutes using a 150 W xenon lamp (Ushio UXL-159) with a monochromator. While no change in the spectra was observed for Ap3, prominent time-dependent changes were observed for FM4-64.

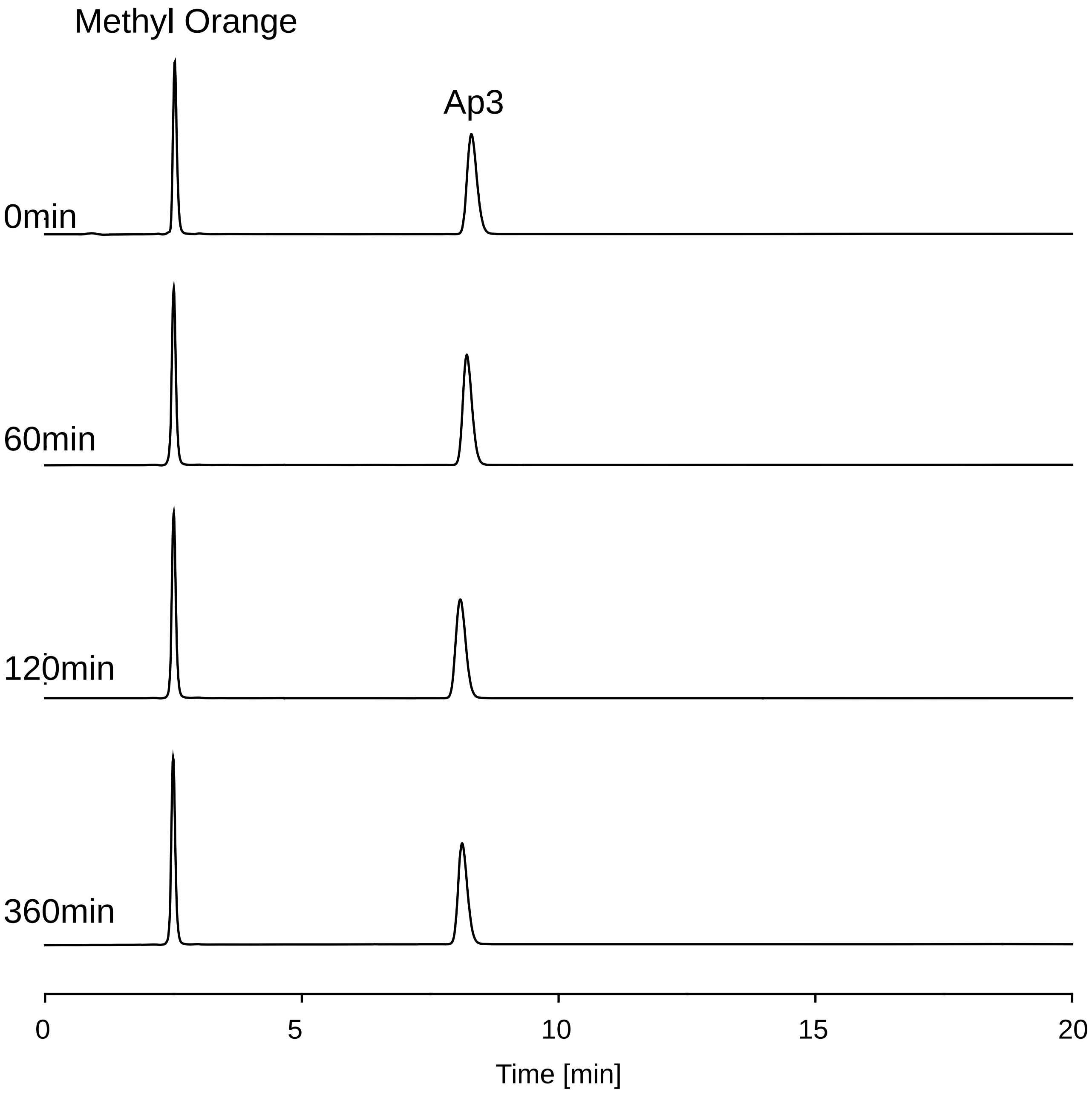

Supplementary Figure 3: Photostability of Ap3 assessed by HPLC.

HPLC chromatograms of Ap3 at irradiation times of 0, 60, 120, and 360 minutes after photo-irradiation. Methyl orange was used as an internal standard (left peak). No new peak was observed during the course of the photo-irradiation.

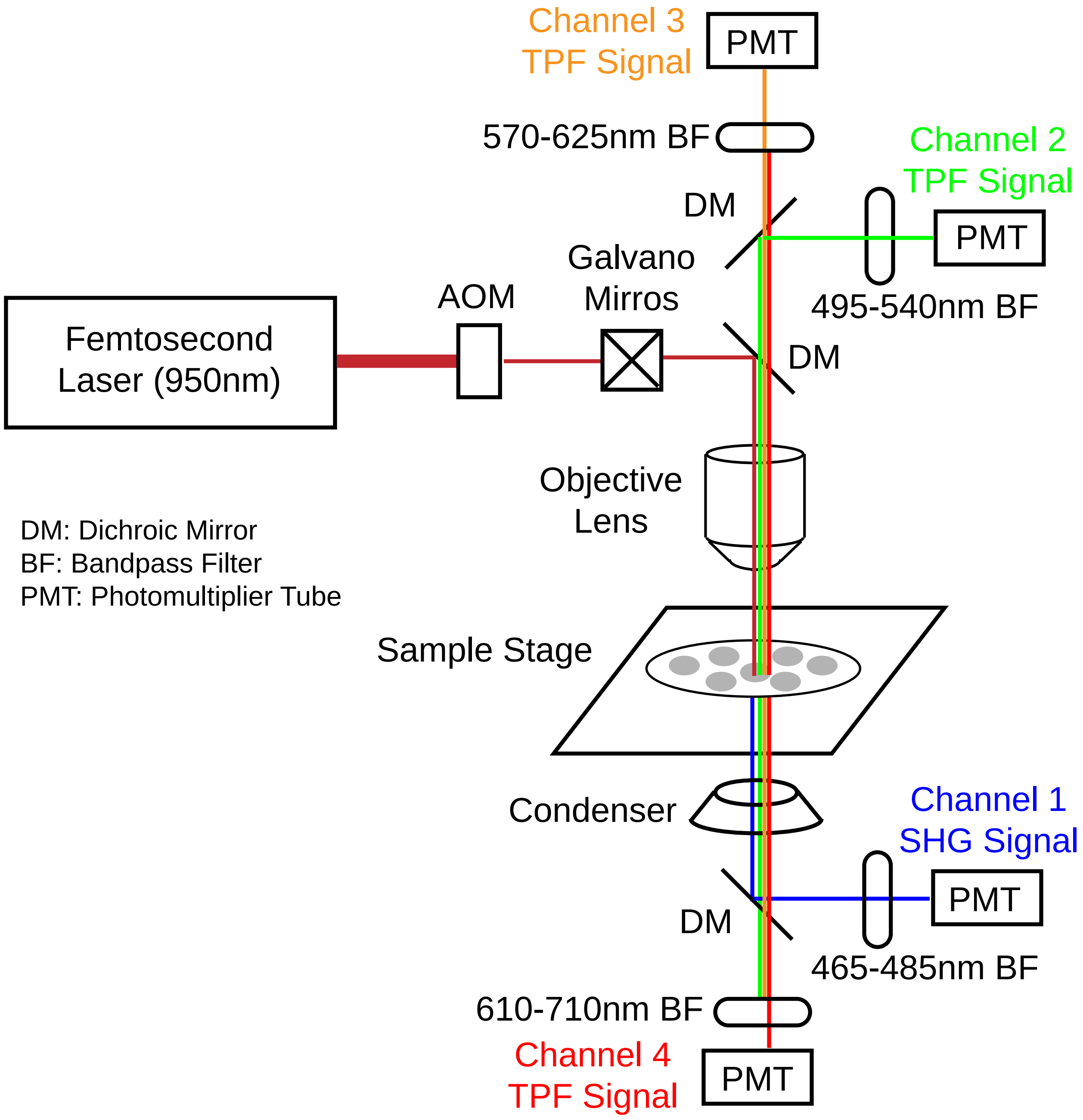

Supplementary Figure 4: Schematic diagram of the experimental setup.

SHG and TPF signals upon 950 nm laser illumination were collected by four external photo-multiplier tubes (PMTs) after band pass filters using the Olympus FV1000MPE multi-photon microscopy system .

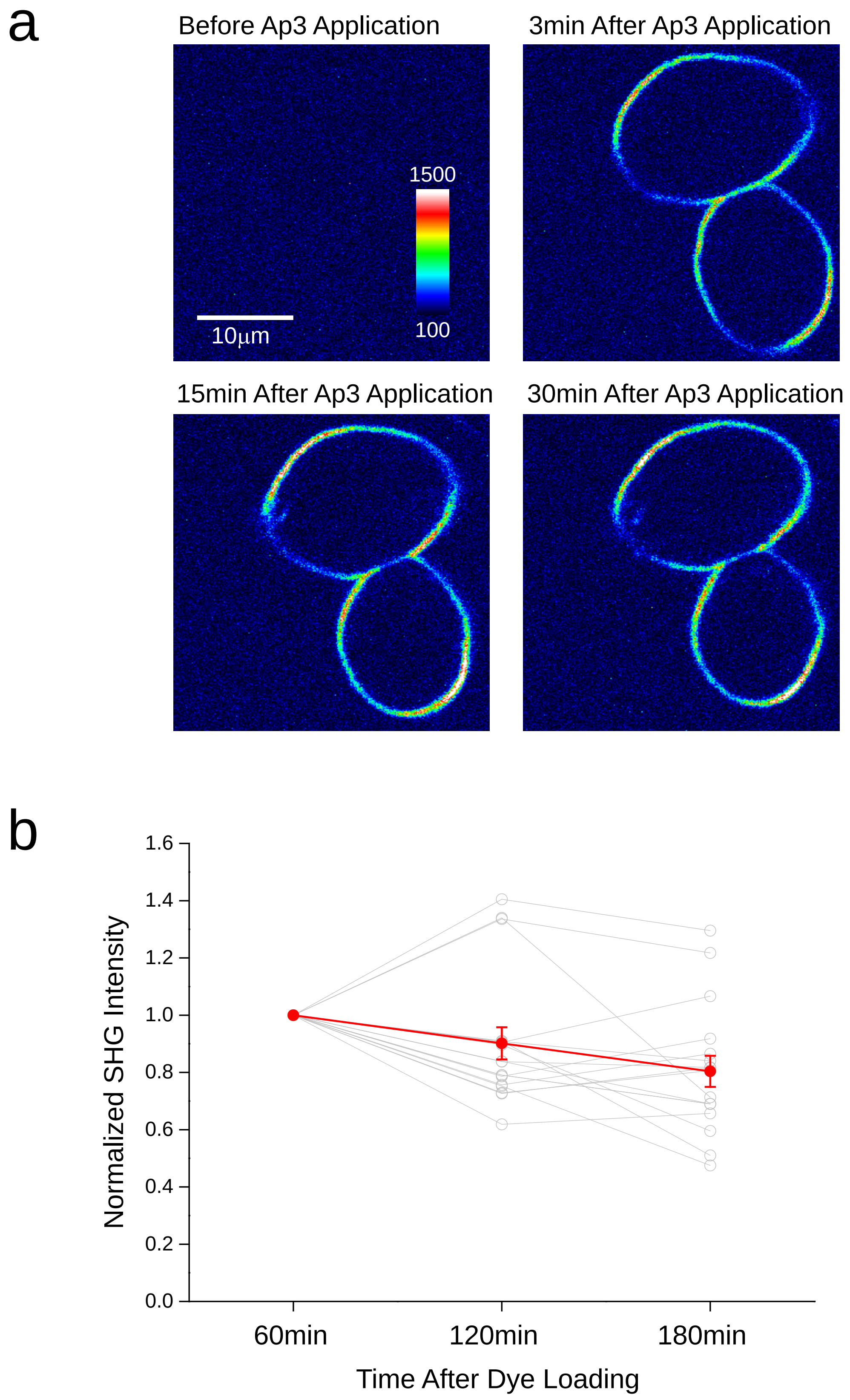

Supplementary Figure 5: Time-course of Ap3 staining.

(a) Rapid adsorption of Ap3 to CHO cells. SHG images were collected from CHO cells before and 3, 15, and 30 min after the application of Ap3.

(b) Gradual decrease in SHG signals after loading. SHG signals were collected from CHO cells incubated with Ap3 for 60, 120, and 180 min. Data from individual cells (gray, $n = 17$) as well as the mean ± standard error of the mean (red) are shown. $p < 0.002$ (120 min and 180 min compared to 60 min) by the Steel-Dwass test after the Friedman ANOVA.

a

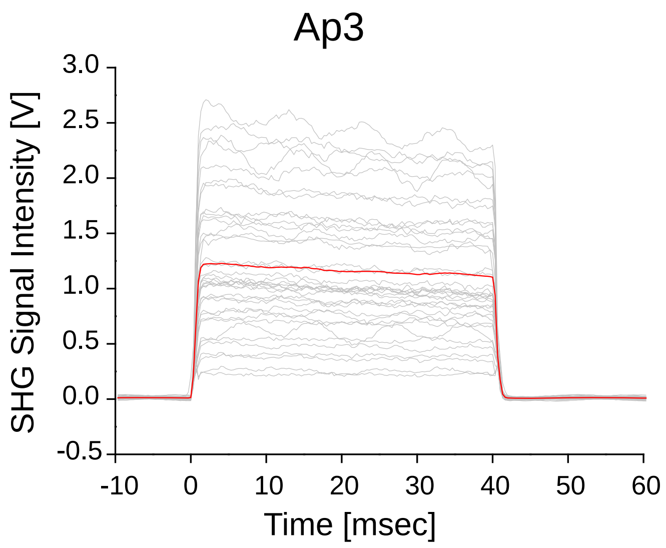

b

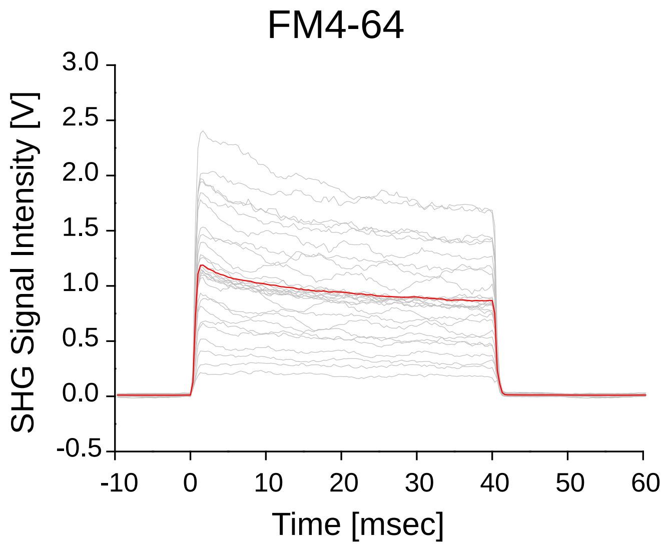

Supplementary Figure 6: Photostability of Ap3 and FM4-64 assessed by point-scan recordings in neurons.

SHG signals during 40 msec-long pointscan measurements obtained from neurons loaded with Ap3 (a, n = 38) and FM4-64 (b, n = 28). Traces of individual recordings (gray) as well as average signals (red) are shown.

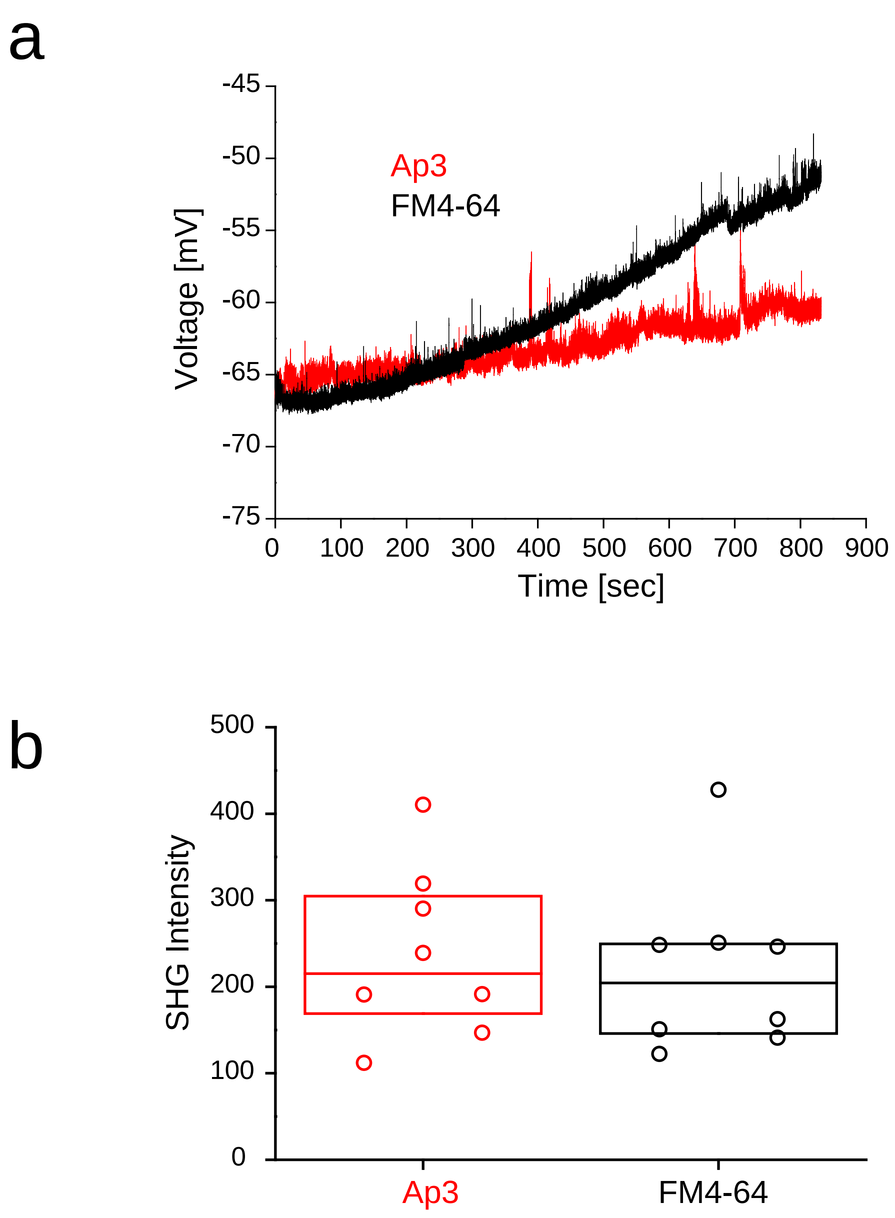

Supplementary Figure 7: Phototoxicity of Ap3 and FM4-64 assessed by slow frame-scan imaging in neurons.

(a) Representative voltage traces of neurons loaded with 500 µM Ap3 (red) and 200 µM FM4-64 (black) during slow frame-scan imaging.
(b) SHG signal intensities of neurons loaded with 500 µM Ap3 and 200 µM FM4-64, which were used for phototoxicity analyses. There are no significant differences in SHG signal intensities between the two groups ($p = 0.79$, $n = 8$ for both groups, Mann-Whitney test).

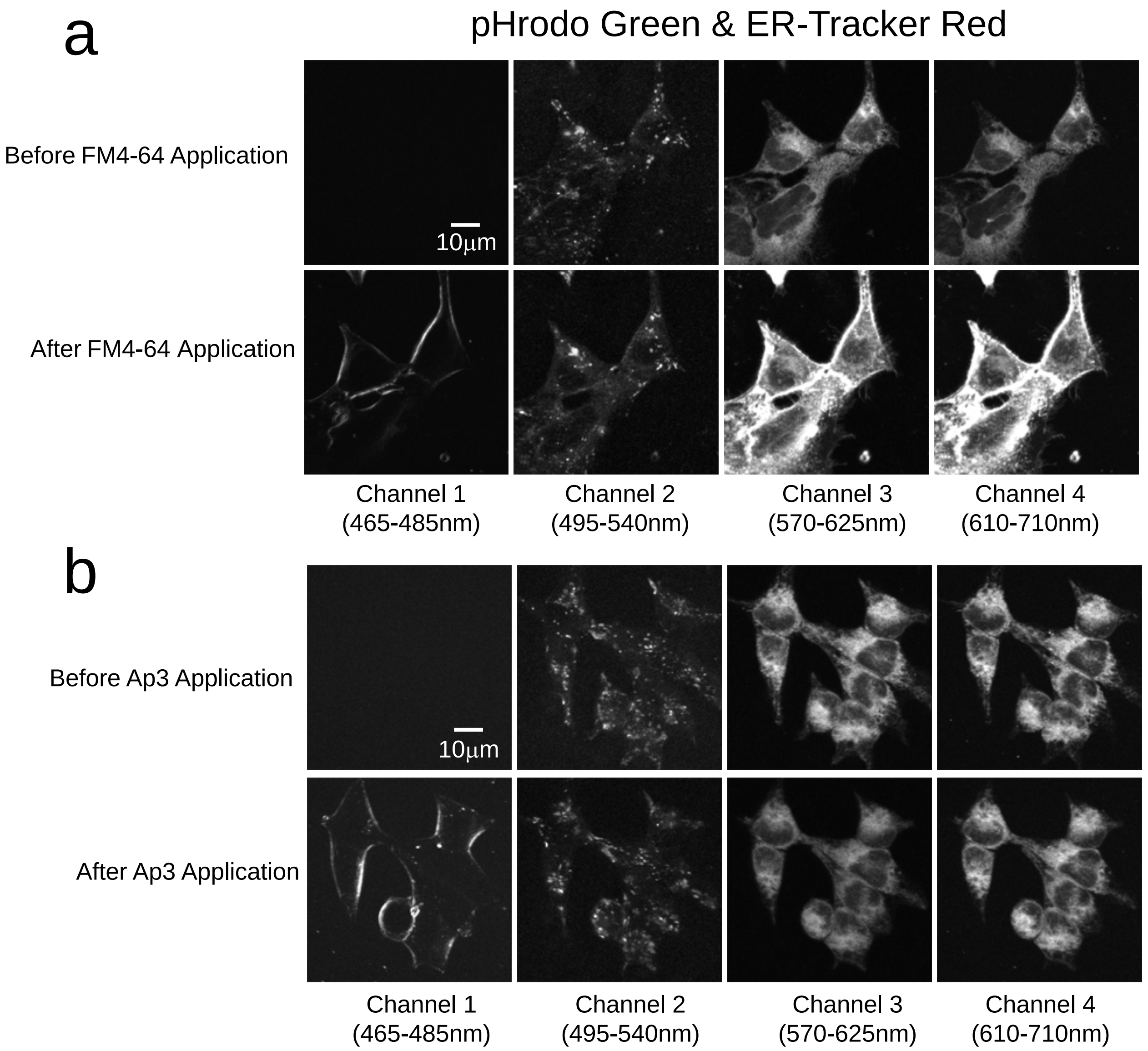

Supplementary Figure 8: Multimodal two-photon imaging with pHrodo Green and ER-Tracker Red.

CHO cells were loaded with pHrodo Green coupled to 10 kDa dextran and ER Tracker Red and visualized before and after the application of FM4-64 (a) or Ap3 (b) using a 950 nm laser.

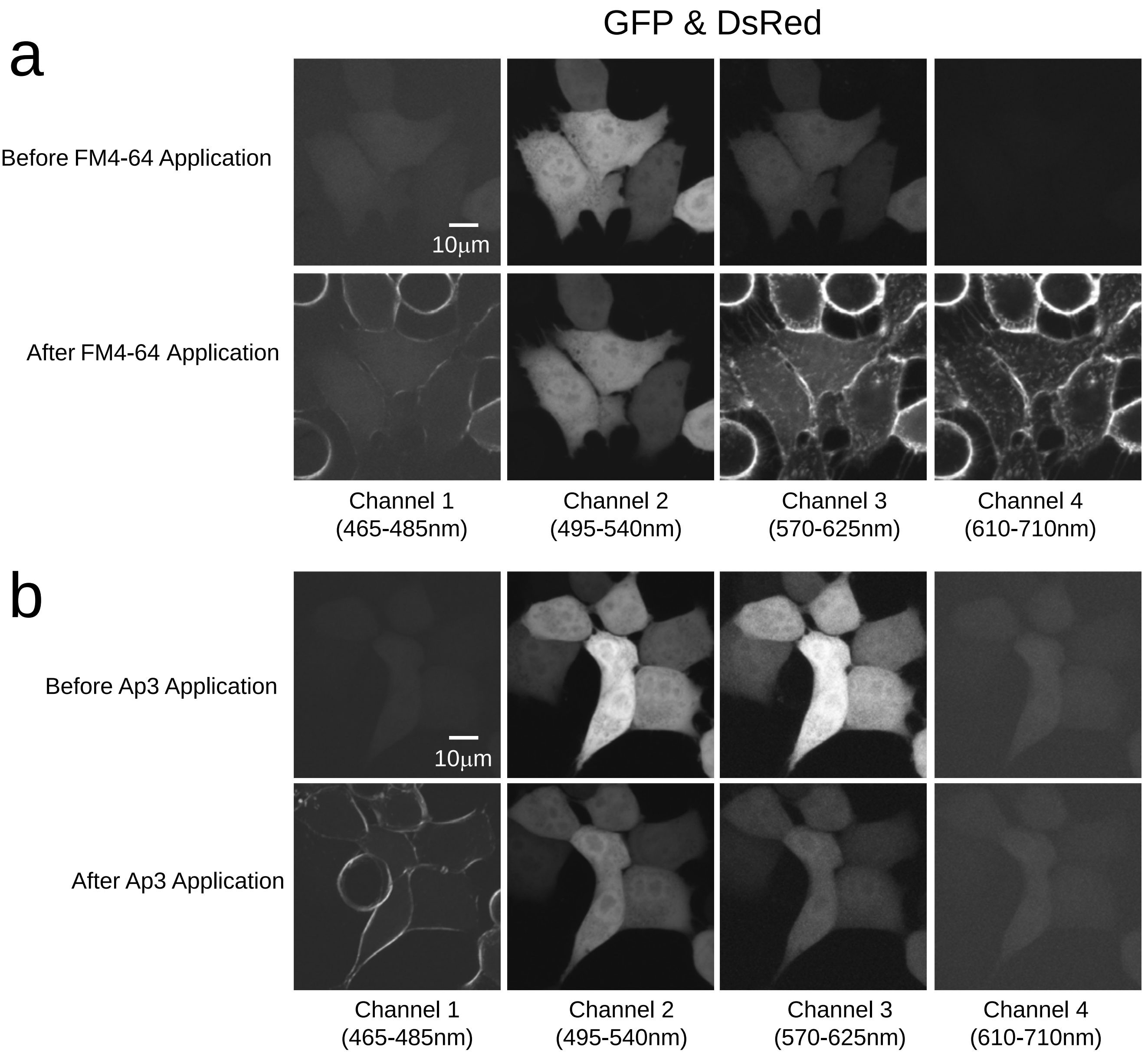

Supplementary Figure 9: Multimodal two-photon imaging with EGFP and DsRed.

CHO cells were transfected with pEGFP-N1 and pDsRed-N1 empty vectors and visualized before and after the application of FM4-64 (a) or Ap3 (b) using a 950 nm laser.

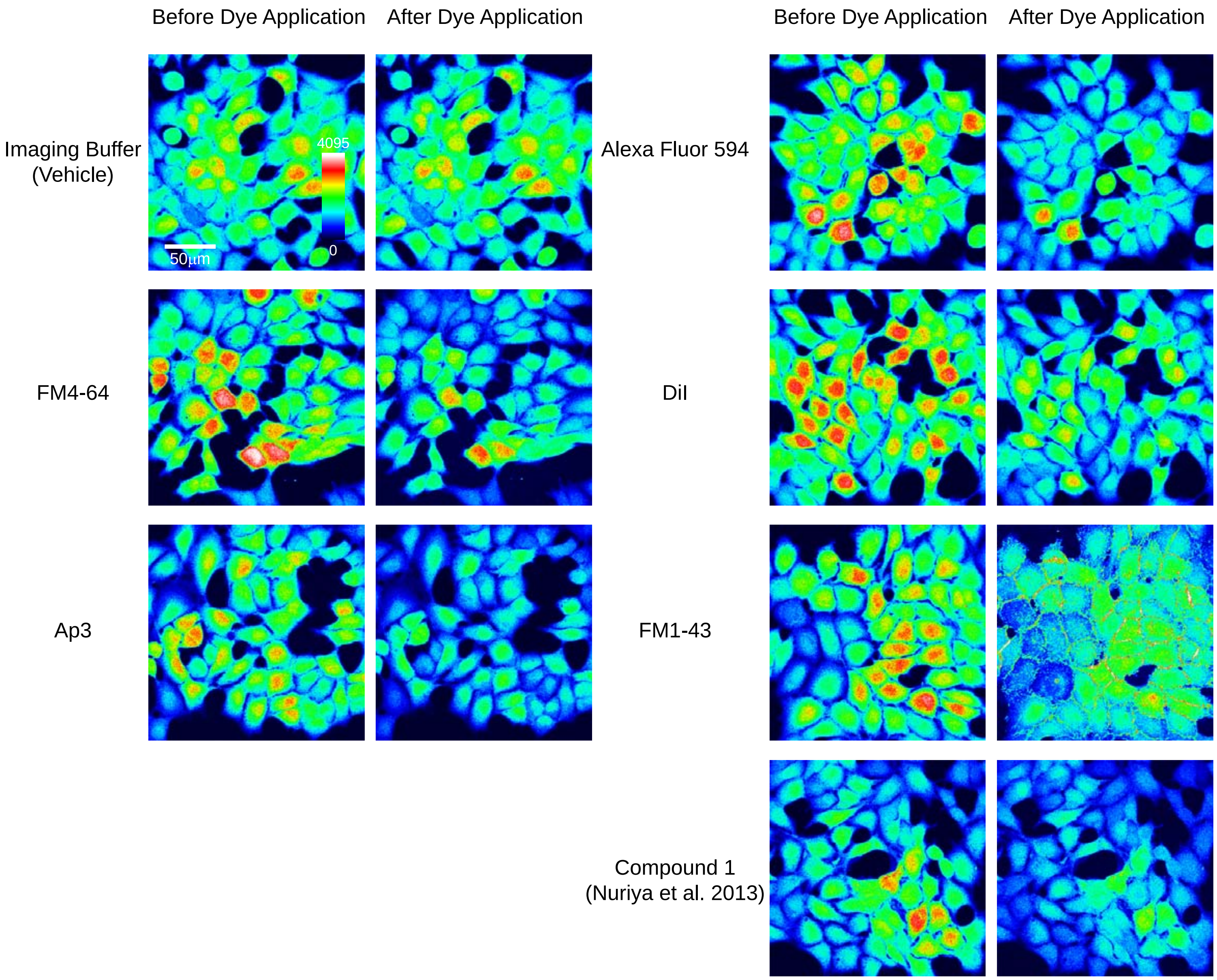

Supplementary Figure 10: Reduction of fluorescence signals following the introduction of chromophores.

CHO cells were loaded with 500nM Calcein and the fluorescence signals were monitored in the whole field of view before after the addition of the other chromophores (FM4-64, Ap3, Alexa Fluor 594, DiI, FM1-43, and Compound 1 in Nuriya et al. 2013, all at a final concentration of 20 μM).

Alexa Fluor 488 and Rhod-2 Only (No SHG Dye)

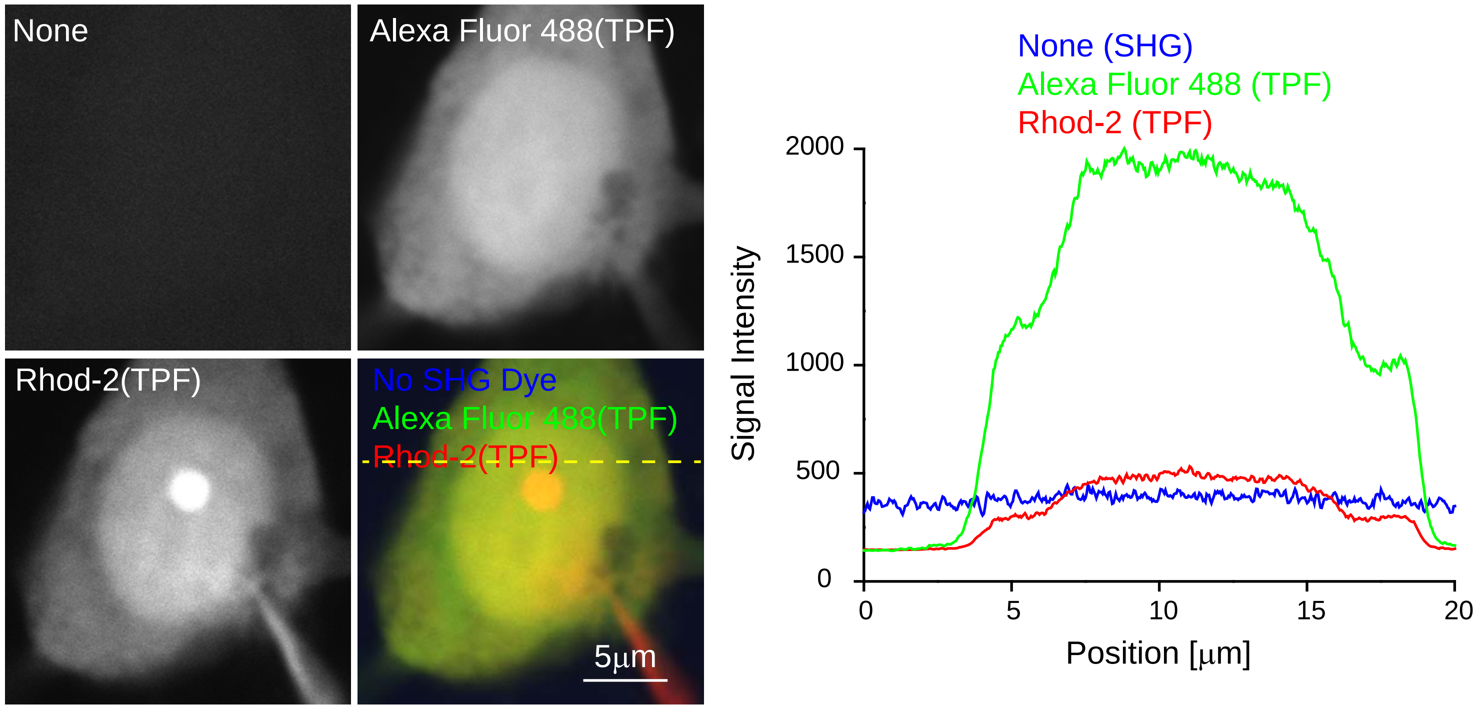

With FM4-64

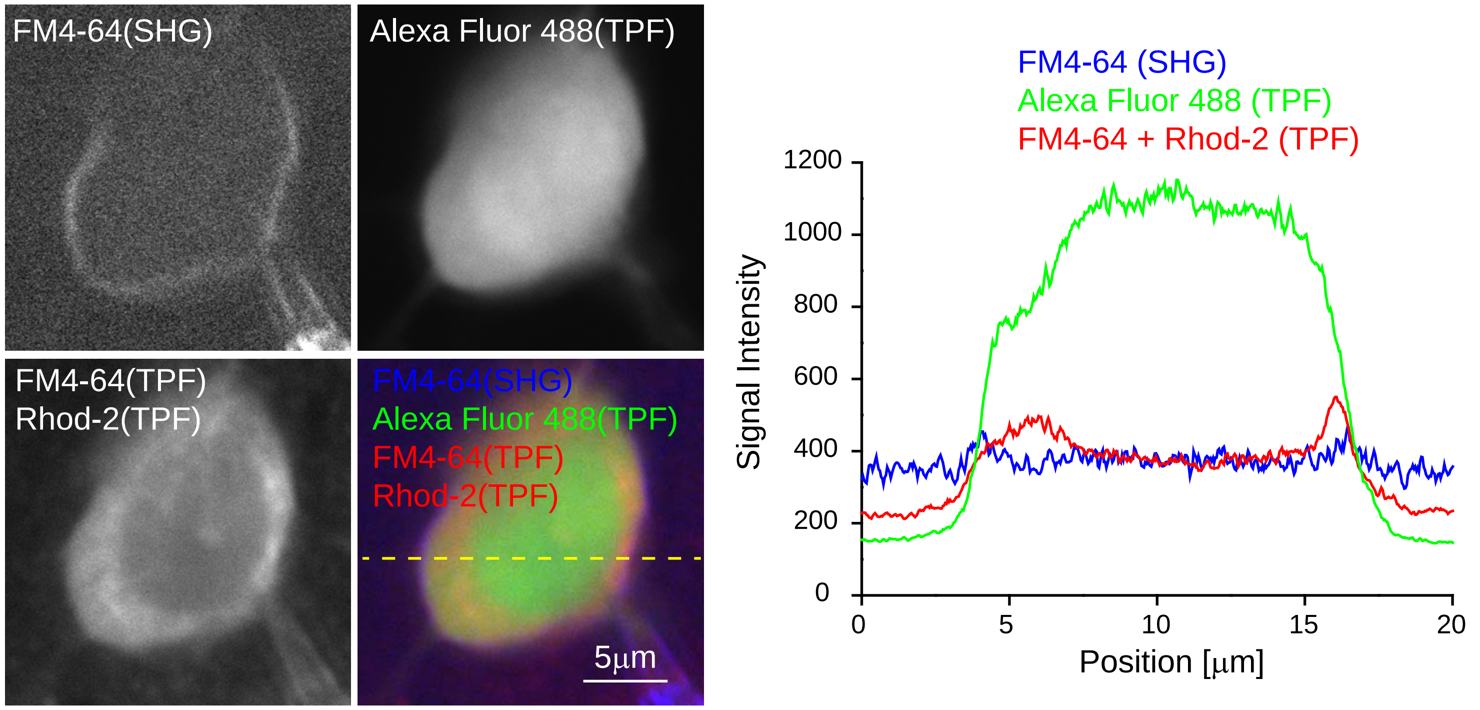

Supplementary Figure 11: Multimodal two-photon imaging of Alexa Fluor 488 and Rhod-2 with or without FM4-64.

Neurons were loaded with 100 μM Alexa Fluor 488 and 100 μM Rhod-2, without (top) or with 100 μM FM4-64 (bottom). TPF signal of FM4-64 dominates that of Rhod-2 and significantly alters the fluorescence intensity profile at the lines indicated.

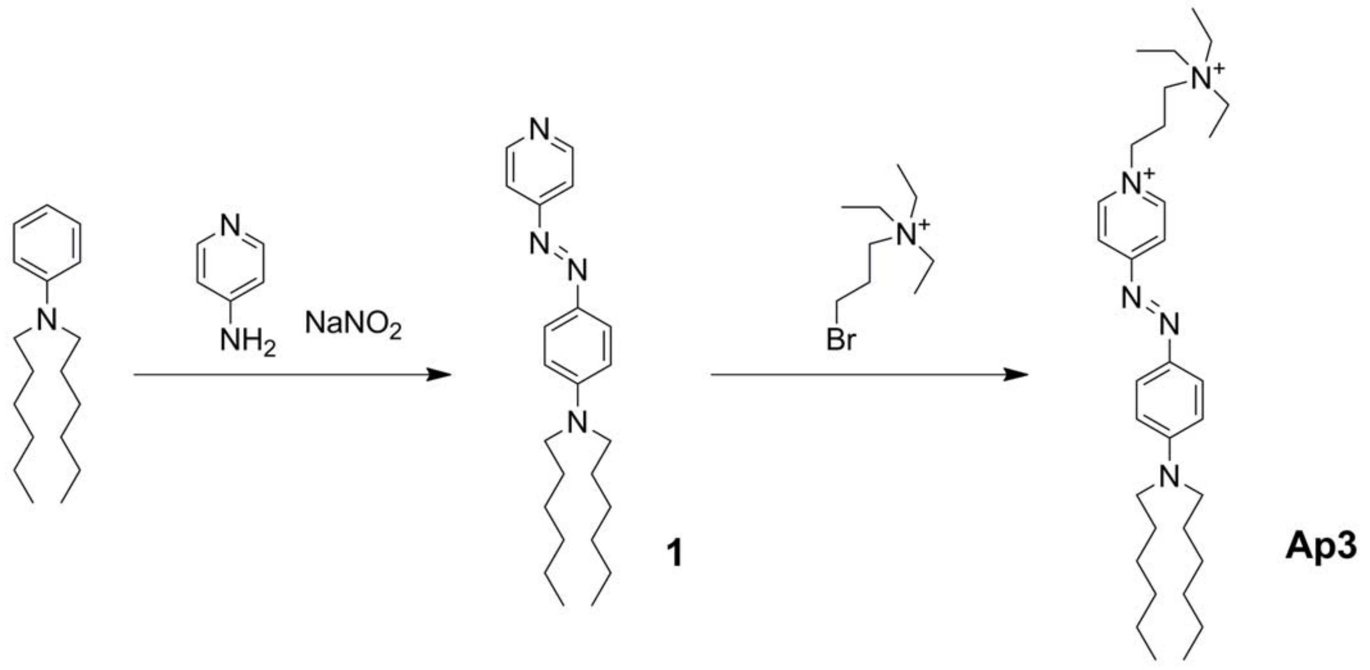

Supplementary Figure 12: Schematic illustration of the chemical sysnthesis of Ap3.
