# Supplementary material for: Multimodal two-photon imaging using a second harmonic generation-specific dye
Source: Nat Commun. 2016 May 9;7:11557. doi: 10.1038/ncomms11557 (PMC4865818; doi:10.1038/ncomms11557)
Supplement: Peer review file [file ncomms11557-s2.pdf]

Transferred manuscripts:

Editorial Note: this manuscript has been previously reviewed at another journal that is not operating a transparent peer review scheme. This document only contains reviewer comments and rebuttal letters for versions considered at Nature Communications.
